# Performance and Material-Dependent Holistic Representation of Unconscious Thought: A Functional Magnetic Resonance Imaging Study

**DOI:** 10.3389/fnhum.2019.00418

**Published:** 2019-12-06

**Authors:** Tetsuya Kageyama, Kelssy Hitomi dos Santos Kawata, Ryuta Kawashima, Motoaki Sugiura

**Affiliations:** ^1^Department of Human Brain Science, Institute of Development, Aging and Cancer, Tohoku University, Sendai, Japan; ^2^Center for Evolutionary Cognitive Sciences, Graduate School of Arts and Sciences, The University of Tokyo, Tokyo, Japan; ^3^Department of Advanced Brain Science, Institute of Development, Aging and Cancer, Tohoku University, Sendai, Japan; ^4^Department of Ubiquitous Sensing, Institute of Development, Aging and Cancer, Tohoku University, Sendai, Japan; ^5^Department of Disaster-Related Cognitive Science, International Research Institute of Disaster Science, Tohoku University, Sendai, Japan

**Keywords:** unconscious thought, fMRI, holistic representation, multimodal function, decision-making

## Abstract

Psychological research has demonstrated that humans can think unconsciously. Unconscious thought (UT) refers to cognitive or affective decision-related processes that occur beyond conscious awareness. UT processes are considered more effective in complex decision-making than conscious thought (CT). In addition, holistic representation plays a key role in UT and consists of a multimodal, value-related cognitive process. While the neural correlates of UT have recently been investigated, the holistic representation hypothesis of UT has not been confirmed. Therefore, in the present study, we aimed to further evaluate this hypothesis by utilizing two UT tasks (person and consumer-product evaluations) in conjunction with an improved functional magnetic resonance imaging (fMRI) experimental protocol. Participants evaluated four alternatives with 12 attributes each. In the UT condition, once the decision information had been presented, the participants completed a 1-back task for 120 s and evaluated each alternative, as well as an independent 1-back task in the absence of any decision information. We then performed regression analysis of the UT performance in both tasks. Our results revealed a positive correlation between performance in the UT task and the use of the anterior part of the precuneus/paracentral lobule in the person evaluation task and between performance and the posterior part of the precuneus, postcentral gyrus, middle occipital gyrus, and superior parietal lobule in the consumer-product evaluation task. The involvement of the precuneus area in both tasks was indicative of a multimodal, value-related process and is consistent with the features of holistic representation, supporting a central role for holistic representation in UT. Furthermore, the involvement of different precuneus subregions in the two UT tasks may reflect the task dependency of the key representation critical for advantageous UT.

## Introduction

Research has revealed that we have the ability to think unconsciously. Unconscious thought (UT) refers to cognitive or affective decision-related processes that occur beyond conscious awareness, i.e., while people are consciously occupied in performing some other task (Dijksterhuis and Nordgren, 2006; Dijksterhuis and Strick, 2016). Research has demonstrated that UT is effective in solving complex decision-making issues. Every day, we face instances that require complex decision-making – from buying expensive things (e.g., a car) to choosing a college to attend or deciding which company to join. Such decisions typically involve a considerable amount of complex information with various dimensions and pros and cons. Most individuals believe that conscious deliberation produces the best decisions in such complex decision-making scenarios. However, previous psychological studies have refuted this idea (Dijksterhuis and Nordgren, 2006). Conscious thought (CT) is of limited use because of its low capacity (Miller, 1956) and overrates the importance of some collateral attributes (Levine et al., 1996).

The advantages of UT over CT in complex decision-making have been recognized as evidence of the existence of UT, as demonstrated by several studies using similar paradigms (Dijksterhuis, 2004; Zhong et al., 2008; Dijksterhuis et al., 2009; Lassiter et al., 2009; Lerouge, 2009; Ham and van den Bos, 2010; Strick et al., 2010; Usher et al., 2011; Abadie et al., 2013; Creswell et al., 2013; Gentile et al., 2013; Reinhard et al., 2013). Typically, participants in such studies are initially presented with information. For example, they are instructed to read information regarding four alternatives, such as cars or roommates. Following this, they are instructed to rate each alternative on the basis of several attributes (typically 12 attributes/alternatives). The participants decide on their evaluation immediately after reading the information, after a certain period of CT, or after a period of UT during distraction. In other studies, participants performed a distractor task, such as an *n*-back task (Bos et al., 2008) or an anagram task (Dijksterhuis, 2004), to prevent CT. These studies showed that in complex and multifaceted decision-making scenarios, participants who are distracted show better decision-making performance compared with those who make an immediate decision (ID) and are allowed to consciously think regarding their choices.

A holistic representation of decision information is a multimodal, value-related cognitive process that may be associated with larger UT effects. Research has demonstrated that a holistic, non-feature-based mental representation of each decision alternative in UT results in improved decision-making; i.e., holistic processing produces a large UT effect. For example, Lerouge (2009) reported that UT works better when participants holistically process decision information compared with when they process feature-based rating of alternatives. Presenting decision information in blocked choice per option leads to improved decision-making once the distraction period is over (Abadie et al., 2016), and it has a larger UT effect compared with random presentation of decision information, because it stimulates the integration of information into evaluatively coherent representations (Strick et al., 2011). Holistic representation is considered an abstract or higher-order product of the integration of multiple pieces of concrete information (Bos and Dijksterhuis, 2011) and is therefore comparable with a multimodal representation generated by integrating multiple unimodal inputs in a hierarchically organized cognitive system (Mesulam, 1998). Moreover, some researchers assume bottom-up and top-down processes of impression formation (Bos and Dijksterhuis, 2011). Individuating information is a bottom-up process, whereas depending on a previously activated schema or stereotype is a top-down process. UT follows a bottom-up process (Dijksterhuis and Nordgren, 2006). Multimodal mental imagery is majorly UT, and several experimental findings of multimodal mentality are bottom-up processes (Nanay, 2018). Holistic representation is considered relevant in either assessing importance (Bos et al., 2011; Usher et al., 2011) or weighing information (Dijksterhuis and Strick, 2016) to reach an objective summary judgment (Dijksterhuis and Nordgren, 2006), which clearly indicates that holistic representation is value-oriented. Therefore, the holistic representation of UT implies the integration of value information, which is a multimodal cognitive process.

Creswell et al. (2013) performed an experiment to elucidate the neural mechanism of UT and provided the first neuroimaging findings. The stimuli in the experiment were consumer products, including cars, massage chairs, and apartments, which were used as described by Dijksterhuis et al. (2006). Each decision alternative included 12 attributes. Under UT conditions, participants engaged in a 2-back distractor task after being presented with the alternatives; the same 2-back task without alternative presentation (i.e., UT) served as the control condition. The findings revealed a greater involvement of both the right dorsolateral prefrontal cortex (DLPFC) and left middle occipital gyrus during UT. Moreover, the substantial neural activation in both these regions was associated with improved decision-making performance during UT. The authors implied that coordinated neural activation occurs in the DLPFC and left middle occipital gyrus, indicating an unconscious visual and semantic processing of decision information.

However, these findings and their interpretations are inconsistent with the hypothesis that UT operates on holistic representation. Typically, DLPFC is considered to be involved in “conscious” executive functions (Bunge et al., 2001), and the occipital gyrus is considered to be involved in visual processing; both regions have rarely been implicated in multimodal, value-related processing. Research has indicated neural activation of multimodal cortices such as medial prefrontal cortices (Mitchell, 2009) and the precuneus (Krause et al., 1999; Cavanna and Trimble, 2006; Hassabis and Maguire, 2009; Summerfield et al., 2010; Tanaka and Kirino, 2016), relevant to holistic representation. For the value processing of UT, studies have reported neural activation of the ventromedial prefrontal cortex, ventral striatum, and posterior cingulate cortex (Bartra et al., 2013; Clithero and Rangel, 2013). The ventromedial prefrontal cortex plays a role in subjective (Kable and Glimcher, 2007) and emotional (Winecoff et al., 2013) values, whereas the ventral striatum (Knutson et al., 2005; Preuschoff et al., 2006) and posterior cingulate cortex (Gläscher et al., 2008) are involved in reward value.

Creswell et al. (2013) did not identify neural activation consistent with holistic representation; this may be attributed to their research design. First, they used three different consumer products. Holistic representation, which is involved in decision-making, may differ according to the function of each alternative that is evaluated. Areas of the brain within the multimodal association cortices that represent the decision-relevant, multimodal, value-related cognitive process may vary across cars, massage chairs, and apartments. Second, the UT effect depends on the method of decision alternative presentation. In the study by Creswell et al. (2013), participants were serially shown the attributes for alternatives in random order. To gain neuroscientific evidence of the holistic representation of UT, the decision information should not be presented randomly. Third, the distraction task influences the results. The study used a 2-back task. However, several experiments have demonstrated that, compared with a more taxing distraction task, a relatively light distraction task leads to better UT results (Dijksterhuis and Strick, 2016). For these three reasons, Creswell et al. (2013) were unable to identify the neural correlates of holistic representation with sufficient sensitivity.

Therefore, in the present study, we have focused upon these three potential issues and improved the experimental design them to identify the neural correlates of holistic representation in UT. First, to clarify the value-related cognitive process, we used a person evaluation task as a stimulus. One type of consumer product does not include numerous attributes related to its value; hence, we cannot obtain multiple stimuli. However, the value of a person has several attributes and can provide numerous stimuli. Previous psychological research has demonstrated the advantage of UT in person evaluation tasks (Dijksterhuis, 2004; Bos et al., 2008; Strick et al., 2010; Usher et al., 2011). In our study, to compare the differences between tasks, we conducted a consumer-product evaluation task using the same three consumer products (cars, massage chairs, and apartments) as described in the study by Creswell et al. (2013). In addition, to encourage holistic representation, we presented decision information in blocked choice per option. Finally, we used a 1-back task as a distractor task. In addition, because a recent study (Nieuwenstein et al., 2015) has shown that a significant UT effect was not obtained in the average of the subjects, we supposed that the UT effect likely has huge individual differences. Therefore, we conducted a simple condition difference analysis as well as a regression analysis correlated with UT performance. We aimed to assess whether the UT advantage is associated with activation in the multimodal, value-related representation in the brain, as expected from the holistic representation hypothesis. We anticipated increased neural activation in the cortical midline structure relevant to better UT performance, which is related to the multimodal, value-related cognitive process.

## Materials and Methods

### Participants

We enrolled 34 healthy right-handed students (19 males and 15 females; age range, 18–25 years) (Oldfield, 1971) at Tohoku University (Japan) in the study. All participants had normal or corrected-to-normal vision, with no history of neurological or psychiatric illnesses. The study protocol was approved by the ethics committee of the Tohoku University School of Medicine, and each participant provided written informed consent before the study. Data on eight participants in the person evaluation task and four in the consumer-product evaluation task were excluded owing to large head movements (>4 mm) during functional magnetic resonance imaging (fMRI).

### Stimuli

**FIGURE 1:**
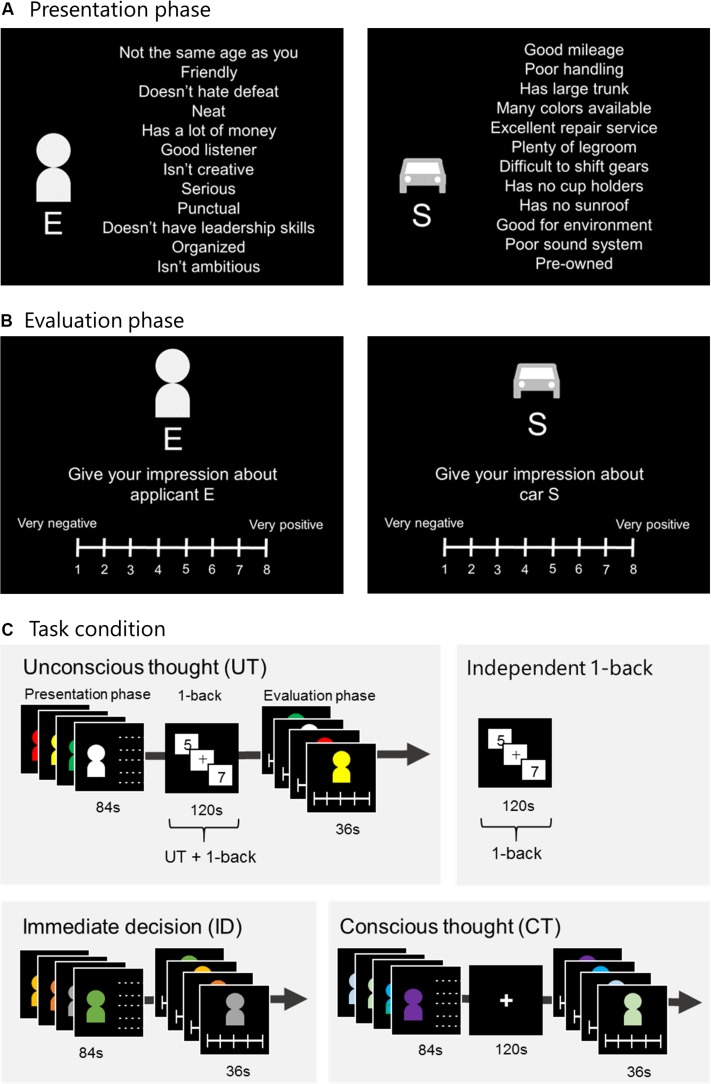
Experimental task. **(A)** Presentation phase: Alternatives E–H were presented in a random order for 21 s each. **(B)** Evaluation phase: Participants rated each alternative within 9 s using an 8-point Likert scale. **(C)** Under UT conditions, participants engaged in a 1-back task for 120 s between the presentation and evaluation phases. Under ID conditions, the evaluation phase was immediately followed by the presentation phase. Under CT conditions, after presenting the alternatives, participants were given 120 s to think carefully. The independent 1-back task served as the control task for UT, and participants were instructed to engage in the task. UT, unconscious thought; CT, conscious thought; ID, immediate decision.

The procedures followed conformed to those reported by Creswell et al. (2013). Each decision alternative was represented by a pictogram of either a human figure or a consumer product, along with 12 attributes (Figure 1A). In the person evaluation task, the decision context was modified from a study by Strick et al. (2010) where participants were asked to rate a new dormitory roommate. However, because it is not common for Japanese university students to choose roommates, for our study, instead of roommates, we used university club members. For the two tasks, participants were instructed to imagine recruiting a new university club member or buying a consumer product and were presented with decision information considering four attributes (one being the most attractive, one being the least attractive, and the remaining two being intermediately attractive). They were asked to form an impression about each one and rate them using an 8-point Likert scale ranging from very positive to very negative (Figure 1B). All attributes of the person evaluation task were finalized using preparatory experiments (Supplementary Material), and those of the consumer-product evaluation task were the same as those used by Creswell et al. (2013). In addition, Creswell et al. (2013) showed their participants 12 attributes serially. However, in the present study, we showed our participants 12 attributes at a time to enhance their holistic impression and UT effectiveness (Lerouge, 2009; Abadie et al., 2016). We were interested in the difference between neural activity during the 1-back task under UT conditions and the independent 1-back task. CT and ID were included for the purpose of comparing task performances.

### Tasks

Each session included three thought conditions (UT, CT, and ID) and one independent 1-back task (Figure 1C). Under UT conditions, after the attributes were presented for 84 s in the presentation phase, participants were distracted with a 1-back task for 120 s. In this 1-back task, participants were serially shown single-digit numbers and instructed to press a button when the current and previous numbers were the same. Thereafter, in the evaluation phase, they were instructed to rate each alternative. Participants indicated their evaluation using an MRI-compatible keypad. Fingers from the left little finger to the right little finger were assigned ratings of 1–8, respectively (ranging from 1 for very negative to 8 for very positive; Figure 1B). In participants who were engaged in the 1-back task, we expected that UT would occur. Under ID conditions, after the alternatives were presented, participants were instructed to rate them immediately. Under CT conditions, after alternatives were presented, participants were instructed to rate them after carefully thinking for 120 s. Overall, three sessions were conducted for the person and consumer-product evaluation tasks. The task order (person or consumer-product evaluation), thought conditions, and the independent 1-back task were randomly assigned to each participant.

### Behavioral Data Analysis

The evaluation performance was calculated using the conventional approach (Dijksterhuis and Nordgren, 2006) – determining the difference in evaluation between the most and least desirable choices. A higher score indicated better decision performance under each thought condition. We conducted one-way analysis of variance (ANOVA) to compare the decision performance under the three thought conditions. Statistical data analysis was conducted using SPSS Statistics version 22 for Windows (IBM Corp., Armonk, NY, United States). A *p*-value of <0.05 indicated statistical significance.

### fMRI Experimental Procedures

First, participants received instructions for the task and performed practice sessions with alternatives that were not used for the fMRI experiment. Thereafter, each participant lay comfortably on the bed of the MRI scanner with their head fixed with foam blocks. All images were rear-projected onto a semi-lucent screen that could be viewed by the participant via a mirror attached to the MRI head coil. The visual angle was set at <5°. Two MRI-compatible button response boxes with four buttons attached to a plastic board were placed on the participant’s abdomen, and each of the eight fingers was properly placed on the corresponding button. Stimuli were presented using PsychoPy ver. V1.84.1^1^ (Peirce, 2007).

### fMRI Data Acquisition

All fMRI data were obtained using a 3T Philips Achieva scanner (Philips Healthcare, Best, Netherlands) using an echo-planar sequence sensitive to the blood oxygenation level-dependent contrast. The parameters were as follows: 64 × 64 matrix; TR = 2500 ms; TE = 30 ms; flip angle = 85°; field of view = 192 mm; and 40 slices with 2.5-mm thickness and 0.5-mm gap. The following preprocessing procedures and statistical data analysis of fMRI data were conducted using Statistical Parametric Mapping (SPM12) software (Wellcome Department of Imaging Neuroscience, London, United Kingdom) and MATLAB (MathWorks, Natick, MA, United States): adjustment of the acquisition timing across slices, correction for head motion, spatial normalization using the EPI Montreal Neurological Institute (MNI) template provided in SPM, and smoothing using a Gaussian kernel with a full-width at half-maximum of 8 mm.

### fMRI Data Analysis

Statistical analyses were conducted using a conventional two-level approach for multiple-subject fMRI datasets. For each session, we separately constructed regressors for the UT phase, independent 1-back phase, CT phase, ID instance, presentation phase (encoding), and evaluation phase. The UT phase occurred between the presentation and evaluation phases, i.e., for 120 s, during which participants performed the 1-back task. The CT phase was also placed between the presentation and evaluation phases, i.e., for 120 s, during which participants evaluated each alternative. The ID instance occurred immediately after the evaluation phase. In addition, regardless of task conditions, we set the duration of the presentation and evaluation phases to 84 and 36 s, respectively. Moreover, we set the unmodeled fixation period as the implicit baseline. We constructed a general linear model for each participant to analyze hemodynamic responses captured by functional images. The general linear model was fitted to each participant’s imaging data. We generated the predicted time course of the fMRI signal by the convolution of the hypothesized neural activity with the hemodynamic response function provided in SPM12. To decrease the effects of low-frequency noise, we applied high-pass filtering with a frequency cutoff at 480 s/cycle. In the first-level analysis, we made a UT > independent 1-back task contrast for all participants.

In this study, we performed four types of group-level random-effect analyses using a similar approach to that described by Creswell et al. (2013). First, we evaluated UT activation instances that were consistent across participants using voxel-wise one-sample *t*-tests for the UT > independent 1-back task. If brain regions showing significant activation were detected, we verified whether the neural activity was correlated with performance in the UT task. Second, we conducted regression analysis of the UT > independent 1-back task using whole-brain voxel-wise analysis, which revealed the brain regions with correlations between performance in the UT task and brain activity for each subject. For individual difference analyses, we used performance in the UT task itself rather than UT-CT as an indicator of UT. In terms of performance, UT-CT includes not only individual differences in UT but also individual differences in CT. Furthermore, conjunction analysis was implemented to test the commonality of brain regions between tasks in the UT phase, ascertaining whether UT is domain-general or domain-specific. Third, we investigated whether the UT and CT processes are similar or different, following the method described by Creswell et al. (2013). For comparison with the results of UT > independent 1-back, we performed CT > fixation in both person and consumer-product evaluation tasks. We performed a regression analysis for CT > fixation for comparison with the regression analysis for the UT > independent 1-back task. The former approach shows differences in neural activities due to thought condition, whereas the latter approach verifies differences in brain regions that correlate with performance in each thought condition. Fourth, we tested the neural reactivation hypothesis proposed by Creswell et al. (2013). According to this hypothesis, neural regions that are active during the encoding period continue to process that information during subsequent distractor tasks. For reference, we performed the conjunction analysis of UT > independent 1-back task and encoding > fixation. This analysis is performed to identify brain regions that are commonly active in UT and encoding. The statistical threshold at each voxel was *p* < 0.005 (uncorrected), which was corrected to the family-wise error of *p* < 0.05 for multiple comparisons using the cluster size.

## Results

### Behavioral Results

The decision performance under UT, CT, and ID conditions was 1.72 ± 2.80, 1.52 ± 2.59, and 1.84 ± 2.60, respectively [mean ± standard deviation] in the person evaluation task and 1.87 ± 2.62, 2.08 ± 3.08, and 2.40 ± 2.67 in the consumer-product evaluation task, respectively. One-way ANOVA results showed that the influences of the three thought conditions were not significant (*p* > 0.05) for either person or consumer-product evaluation tasks.

### Imaging Results

To identify common neural correlates of the UT process across subjects, we conducted voxel-wise one-sample *t*-tests for the UT > independent 1-back task, which were intended to be a replication of Creswell et al. (2013). However, no unique areas were activated during the UT distraction period compared with the independent 1-back task in either the person or consumer-product evaluation tasks. Therefore, neural correlates of the UT process were not identified as a group difference. For behavioral data, if the UT effect was significant across subjects, the relationship with UT performance was inferred.

**FIGURE 2:**
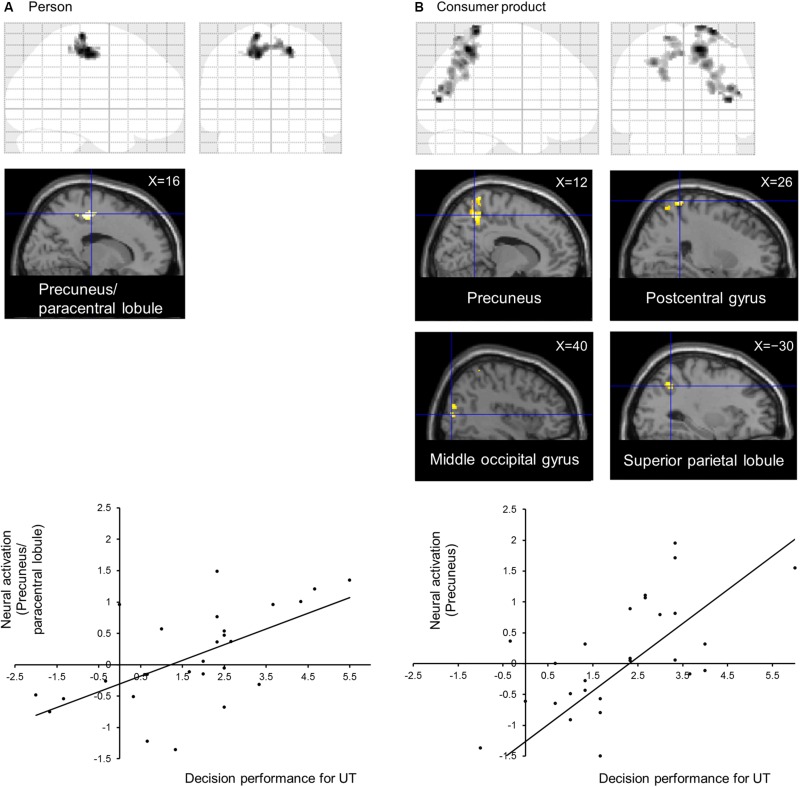
Neural correlates of decision performance under UT conditions in **(A)** person and **(B)** consumer-product evaluation tasks. Brain regions showing task-specific activation were identified using multiple regression analysis. Regional neural activation was superimposed on a sagittal section of anatomical images obtained using SPM12. **(A)** Graph indicating the activation profile for the precuneus/paracentral lobule. **(B)** Graph indicating the activation profile for the precuneus.

The subject average may not be statistically significant because of large individual differences in UT. Therefore, to directly identify the neural correlates of performance in the UT task, we conducted a voxel-wise regression analysis of the performance in the UT task and activation for the UT > independent 1-back task separately for the person and consumer-product evaluation tasks. Our results indicated a significant correlation among separate areas for each task (Table 1). In the person evaluation task, activation spanning the precuneus and paracentral lobule (Figure 2A) was observed, whereas, in the consumer-product evaluation task, the precuneus, postcentral gyrus, middle occipital gyrus, and superior parietal lobule (Figure 2B) were positively correlated with performance in the UT task. The observed brain activation patterns and related cognitive processes were considered to explain performance in the UT task. As expected, these regions consisted of cortical midline structures involved in a multimodal and value-related cognitive process. To identify a common cognitive process related to the performance of the two UT tasks, conjunction analysis of the person and consumer-product evaluation tasks under UT conditions performed. However, our results did not reveal a common activation pattern or process that could explain the performance in the two UT tasks.

**TABLE 1:** Significant neural activity in voxel-wise regression analysis for the UT > independent 1-back task.

**Structure**	**L/R**	**MNI coordinate**			***t***	***k***	***p***
		***x***	***y***	***z***			
**Person evaluation**							
Precuneus/paracentral lobule	R	16	−22	54	3.72	486	0.017
	L	−14	−22	50	3.69		
**Consumer-product evaluation**							
Precuneus	R	12	−54	54	5.12	481	0.011
Postcentral gyrus	R	26	−46	72	4.95		
Middle occipital gyrus	R	40	−82	8	4.76	378	0.035
Superior parietal lobule	L	−30	−56	42	4.12	405	0.026

*MNI coordinates (*x*, *y*, *z*) of the neural activation peak, cluster size (*k*: number of voxels; voxel size = 2 × 2 × 2 mm^3^), and *p* are presented for neural activation in the voxel-wise one-sample *t*-tests for the UT > independent 1-back task in the person evaluation task. Statistical significance refers to *p* < 0.005 at the voxel level (uncorrected) and *p* < 0.05 at the cluster level (FWE-corrected). FWE, family-wise error; MNI, montreal neurological institute.*

For CT > fixation, the voxel-wise one-sample *t*-test was applied to identify neural correlates of the CT process common across subjects. This analysis suggested a significant neural activation of distinct cortical areas for the person and consumer-product evaluation tasks. For the person evaluation task, the insula (*x* = −42, *y* = −4, *z* = 12, and *T* = 5.64; *p* < 0.001) and DLPFC (*x* = −58, *y* = 8, *z* = 24, and *T* = 5.21; *p* < 0.001) were identified. For the consumer-product evaluation task, the ventral occipital cortex (*x* = −18, *y* = −90, *z* = −16, and *T* = 5.71; *p* = 0.012), caudate (*x* = −12, *y* = 10, *z* = −2, and *T* = 5.07; *p* = 0.014), and inferior parietal lobule (*x* = −54, *y* = −20, *z* = 20, and *T* = 5.06; *p* = 0.002) were identified. In addition, we observed that different brain regions were active for the performance of the CT task. In other words, we confirmed the task dependency of CT in this experiment. However, regression analysis of performance in the CT task on the activation of CT > fixation did not reveal any significant activation for either of the tasks. In other words, an activation pattern that could explain performance in each UT task was not identified.

For the conjunction analysis of UT > independent 1-back task and encoding > fixation, which was performed to identify neural correlates of common processes in UT and encoding, no significant activation was observed for either of the tasks. Therefore, our results were not supportive of the neural reactivation hypothesis, which states that the activation of the same area occurs during the encoding and UT distraction period (Creswell et al., 2013).

## Discussion

In this study, we explored the neural correlates of the holistic representation of UT using a single-domain alternative (i.e., person evaluation). We found activation that was correlated with performance in the UT task. Our results suggest that performance is correlated with specific activation during distraction—when UT theoretically operates; this is consistent with the possibility that unique UT processes lead to improved performance. The advantage of UT in complex decision-making scenarios is due to its holistic representation, although previous neuroimaging findings have not supported this hypothesis. In the present study, in addition to the single-domain alternative, we presented information in blocked choice per option, and, to obtain a large UT effect, we used a 1-back task as a distractor task. The present study provided the first neuroimaging finding that demonstrates the validity of the cognitive hypothesis that the advantage of UT is its holistic representation. A correlation between neural activity during UT and decision performance was observed, with activity in the precuneus or its adjacent region, in both person and consumer-product evaluation tasks. Our results indicate that the decision performance of UT is explained by the neural activity of this brain region during UT. The cortical midline structure, including the precuneus, is active during multimodal, value-related cognitive processes, which is consistent with the features of holistic representation.

Several studies have supported the role of the precuneus in holistic representation: the precuneus integrates multimodal information collected from various brain regions, thereby playing an important role in mental image processing (Hassabis and Maguire, 2009; Summerfield et al., 2010; Tanaka and Kirino, 2016). These functional characteristics are consistent with the concept of multimodal processing of holistic representation.

Evaluation-relevant holistic representation can vary between decision items. The present study identified no brain regions common to both tasks. Thus, the brain mechanism of UT could be domain-specific. The notion that the UT process is highly task-dependent is supported by a previous finding that the neural basis of creative cognition is highly task-dependent (Dietrich and Kanso, 2010). Moreover, this finding is consistent with the spatially dissociable value representations within the precuneus (Margulies et al., 2009; Bzdok et al., 2015). In this study, strictly, decision performance under UT conditions was positively correlated with the activity in the anterior part of the precuneus/paracentral lobule in the person evaluation task and with that in the posterior part of the precuneus in the consumer-product evaluation task. In the person evaluation task, the positive correlation might holistically represent the social quality of the individual, who is relevant to the interpersonal relationship. Reportedly, the anterior part of the precuneus/paracentral lobule is involved in mentalization, such as emotions and feelings (Kross et al., 2009; Morelli et al., 2014; Takahashi et al., 2015). Mentalizing refers to inferring the mental state of others. An individual’s mental state determines their actions, and it is important for us to be able to accurately understand the minds of others because several inferences are automatically made without any thought or deliberation (Frith and Frith, 2006). In addition, the anterior part of the precuneus/paracentral lobule is activated when we consider our own preferences, not when thinking of others’ preferences (Seger et al., 2004). This brain region is related to affective aspects of social cognition, such as empathy (Hooker et al., 2010) and social exclusion (Bolling et al., 2011a, b). Therefore, in the present study, it was observed that the anterior part of the precuneus/paracentral lobule controlled holistic representation, unconsciously processing the degree of the participants’ likes and dislikes in the social context of choosing university club members. Participants unconsciously made decisions with regard to the four alternatives presented in the person evaluation task.

By contrast, the neural activity of the posterior part of the precuneus correlating with decision performance in the consumer-product evaluation task might holistically represent the hedonic quality of consumer products, which is directly relevant to purchase decisions. The posterior part of the precuneus is related to reward processing (Holliday et al., 2011; Strang and Pollak, 2014; Dinu-Biringer et al., 2016). Further, this brain region corresponds to tool use (Hermsdörfer et al., 2007; Macdonald and Culham, 2015). Consumer products are worth their function, in addition to the reward of owning them. Therefore, neural activation may represent a reward for the purchase decision made in the form of the functional aspect of the consumer product bought. The UT phenomenon appears similar in different psychological studies, but investigating the brain mechanism proves that its quality depends on the task being performed.

The neural activity of brain regions outside the precuneus that are correlated with decision performance might be related to the organization of the information presented, creating a three-dimensional image and simulating their use in the consumer-product evaluation task. The postcentral gyrus, considered the primary somatosensory cortex (Webb and Adler, 2016), plays an important role in the time and space integration of visual and tactile stimuli (Gentile et al., 2013). The neural activity observed in the postcentral gyrus might be related to the images of unconsciously using each consumer product. Therefore, the images might be integrated in the brain as if the consumer products imaged are indeed being used. Results regarding the neural activation of the middle occipital gyrus obtained in the present study coincide with the findings reported by Creswell et al. (2013). The middle occipital gyrus plays an important role in the brain’s visual processing. As Creswell et al. (2013) suggested, this brain region unconsciously maintains the visual representation of decision information. The superior parietal lobule is associated with visuospatial perception, including the representation and manipulation of objects (Johns, 2014). It is possible that participants could imagine using the consumer products by building a stereoscopic image of each product from the stimuli presented. Interestingly, these three brain regions correspond to the complex involving mental rotation (Vingerhoets et al., 2002). The brain regions verified in the present study appear to be consistent with this hypothesis.

The discrepancy between our results and those of Creswell et al. (2013) may provide important insights into current issues related to UT. Creswell et al. (2013) did not identify the activation of the cortical midline structure, whereas we did not replicate the activation of the DLPFC. There were two reasons for this: the difference in alternative presentation and the analysis method. In the present study, decision information was presented in blocked choice per option to encourage holistic representation. By contrast, Creswell et al. (2013) showed decision information serially in the presentation phase. When alternatives are serially presented, participants need to process each alternative; this requires a cognitive load of executive function. Information processing might have been related to the assumption of executive function, including working memory (Bunge et al., 2001). This can be interpreted as the cortical midline structure, rather than the DLPFC, being activated because the process of serially organizing each attribute is unnecessary; instead, holistic representation is required. The serial presentation of decision information and the distractor task (1-back vs. 2-back) load in the study by Creswell et al. (2013) were insufficient for holistic representation during UT. Activation of the DLPFC in the previous study might represent a load of working memory because of the serial presentation of decision information. In the present study, we conducted regression analysis for the UT > independent 1-back task using whole-brain voxel-wise analysis, whereas Creswell et al. (2013) conducted the UT > independent 1-back task using an across-subject design. Therefore, they probably overlooked neural activity correlated with decision performance. It was possible to examine the brain network directly related to the UT effect without omission of searches from the entire brain. Compared with the analytical approaches used by Creswell et al. (2013), our approaches were advantageous in this regard.

Creswell et al. (2013) provided important insights into UT research; however, their cognitive hypothesis did not refer to conventional UT research. Creswell et al. (2013) hypothesized that neural reactivation, occurring in the extrastriate and prefrontal regions, explains the manner in which UT improves decision-making. We acknowledge that their cognitive hypothesis and neural evidence were independent of – or even potentially contradictory to – the theoretical framework of the accumulated psychological literature on the UT process. Their neural reactivation hypothesis, supported by neural evidence (i.e., activation of the visual and dorsolateral prefrontal cortices), appears rather to conform to the feature-based top-down characteristics assumed for the CT process (Milham et al., 2003; Dijksterhuis and Nordgren, 2006) in the previous psychological literature on UT. By contrast, our results are consistent with the conventional framework of holistic representation in the psychological literature.

The present research provides evidence for a psychological UT mechanism. Distinguishing between common UT and advantageous UT processes might be more important. As shown by the several behavioral experiments as well as by our regression analysis, a large UT advantage is caused by blocked presentation and potentially involves holistic representation in multimodal, value-related networks. By contrast, in the UT task, a feature-based top-down process may be involved, particularly in the case of serial presentation, as shown by Creswell et al. (2013). This finding may help in understanding the psychological UT mechanism. Regarding CT, the voxel-wise one-sample *t*-test for CT > fixation indicated significant neural activation in both person and consumer-product evaluation tasks, but the regression analysis for CT > fixation did not give a significant result. We considered the discussion of the former results irrelevant because we did not observe significant findings in UT > independent 1-back for comparison, and activation in CT > fixation itself reflected the various non-specific cognitive processes involved in both tasks. Although we were unable to discuss further regarding the CT mechanism in the present research, we were able to update the psychological mechanism of UT.

It may be important to note that we failed to replicate the average UT advantage across all participants. Research has demonstrated that there is room for reconsideration in UT behavior data. For example, Acker (2008) replicated the study by Dijksterhuis et al. (2006) under the same UT conditions as were used by Creswell et al. (2013) and noted that UT conditions did not improve decision performance compared with the other conditions. Therefore, Nieuwenstein et al. (2015) performed the same experiment using approximately 400 participants, which is approximately 10-fold more than other UT-related studies, and reported that UT conditions did not lead to the most desirable choice compared with the other conditions. However, importantly, considering that these studies did not report significantly lower performance under UT than that under CT conditions, it is highly likely that some UT process occurs to attain a similar level of decision performance as that observed under CT conditions. UT may exhibit substantial individual differences, like ID and CT, and brain measurement could be the main method for its measurement. In psychological research, the existence of UT is denied unless a significant difference is obtained. Furthermore, in this research field, it is debated whether the UT task indeed guarantees that the individual’s thinking process is unconscious (Newell and Shanks, 2014; Garrison and Handley, 2017). However, on examining the relationship with brain activity, as shown in the present study, the correlation between the UT effect and the brain activity of the individual reveals the neural basis of UT as well as possibly proving that UT exists for some individuals. If the UT effect is merely a noise of the behavior parameter, no correlation with significant brain activity can be obtained. There are individual differences between ID and CT, and because these are rational–experiential-thinking styles, we can measure them using the Rational–Experiential Inventory developed by Epstein et al. (1996). However, although it is difficult to measure UT using questionnaires, we can measure unconscious psychological processes using brain measurements. The fact that we identified individual differences in UT performance using brain measurements in the present study demonstrates that there is a brain index for the evaluation of individual differences in UT.

Use of different types of decision items can help reveal the entire brain mechanism of UT in UT study. Decision-making research has advanced by the traditional two-level approaches of intuition and rationality (Kahneman, 2011). Therefore, commonly, we only use either of the two thinking modes – ID and CT – for decision-making. Classically, only ID and CT were studied, and UT recently became a subject of research. The present study demonstrated the potential importance of the quality of UT. The neural activation of UT differs on the basis of task. The effect of UT is observed in other decision-making scenarios, such as lie detection (Reinhard et al., 2013), responses to client requests (Abadie et al., 2013), moral situations (Ham and van den Bos, 2010), and product satisfaction improvements (Messner and Wänke, 2011). Although these studies proved the effectiveness of UT, the brain mechanism in each could be different. In the future, UT can be studied in various research fields such as management or political science. It is anticipated that functional neuroimaging studies will reveal the mechanism of the unconscious decision-making process and will lead to fruitful progress in decision-making research.

One limitation of the present study was a methodological limitation common to other UT studies. In this study, the attributes of desirable university club members or consumer products were based on the element that would be desirable for most individuals. Therefore, the optimal choice was defined by the option having the highest number of positive qualities. However, this method is not reasonable. An option with numerous positive qualities is not always the best option for everybody. For example, even if a car scores well on several attributes, some individuals may not like it because it is a car with manual transmission (González-Vallejo et al., 2008). To advance UT research in the future, changing this methodology might be necessary.

## Ethics Statement

The study protocol was approved by the Ethics Committee of the Tohoku University School of Medicine, and each participant provided written informed consent before the study.

## Author Contributions

TK, RK, and MS designed the research. TK and KS performed the experiments. TK analyzed the data. TK and MS prepared the manuscript. All authors reviewed the manuscript.

## Conflict of Interest

The authors declare that the research was conducted in the absence of any commercial or financial relationships that could be construed as a potential conflict of interest.

## Footnotes

1 http://www.psychopy.org/
