# Supplementary material for: Performance and Material-Dependent Holistic Representation of Unconscious Thought: A Functional Magnetic Resonance Imaging Study
Source: Front Hum Neurosci. 2019 Dec 6;13:418. doi: 10.3389/fnhum.2019.00418 (PMC6908964; doi:10.3389/fnhum.2019.00418)
Supplement: Supplementary file 1 [file Data_Sheet_1.pdf]

*Supplementary Material*

# Performance and Material-Dependent Holistic Representation of Unconscious Thought: A Functional Magnetic Resonance Imaging Study

**Tetsuya Kageyama*, Kelssy Hitomi dos Santos Kawata, Ryuta Kawashima and Motoaki Sugiura**

* **Correspondence**: Tetsuya Kageyama: tkageyama@med.tohoku.ac.jp

## 1. Supplementary Data

### Preparatory experiments for the fMRI experiment

The purpose of the preliminary experiment was to create a person evaluation task for our fMRI experiment. We created 36 imaginary university club members. Participants were presented with four alternatives under three thought conditions (UT, CT, and ID) in each session. We performed three sessions, with three thought conditions in each session. Therefore, 4 (alternatives) × 3 (thought conditions) × 3 (sessions) = 36 imaginary university club members. For creating imaginary university club members, we needed certain attributes. Therefore, we gave each individual 12 personality attributes. Each personality attribute was given a score, and the total score was related to the attractiveness of the individual. Of the 36 imaginary university club members, nine were made very attractive, 18 were made medium attractive, and the remaining nine were made unattractive. We performed two experiments:

First, we conducted a questionnaire survey to find out the attributes that were regarded as important by the Japanese university students when selecting new university club members. In this

experiment, 35 students (26 males, 9 females) with a mean age of 21.1 ± 1.62 years from the Tohoku University (Japan) participated. The personality attributes included, for example, “cooperative,” “calm,” and “has many friends.” Participants rated each attribute using an 8-point Likert scale. On the basis of the total score, nine desirable university club members were selected, having eight positive attributes (e.g., “has high communication skills”) and four negative attributes (e.g., “pessimistic”); 18 intermediate club members were selected, having seven positive and five negative attributes; and nine undesirable club members were selected, having six positive and six negative attributes. Each attribute was scored by averaging the participants’ ratings for that attribute and then summing the scores across all 12 attributes within each alternative. The university club members in each category (desirable, intermediate, and undesirable) had almost the same score in favorability.

Second, we considered stimulus optimization and distractor tasks in this experiment by presenting participants with alphabetical letters or real names (Figure 3). Strick et al. (2010) used real names familiar to the Dutch participants in their roommate choice study. However, using real names could lead to bias. For instance, when participants were shown their friends’ names in the experiment, they imagined specific friends. However, such bias can be avoided by presenting names in the alphabetical order. The names presented in this study were familiar to the Japanese (e.g., Tanaka and Suzuki). The distractor tasks were compared using 1- and 2-back tasks. Participants comprised 15 Tohoku University students (12 males, 3 females) with a mean age of 21.3 ± 1.49 years. We tried three sessions: (i) the 1-back task and an alphabetical letter, (ii) the 2-back task and an alphabetical letter, and

(iii) the 1-back task and a real name. Participants were randomly allocated to the three sessions, and three thought conditions (UT, CT, and ID) were randomly assigned to each participant. We performed ANOVA for the experiment results comparing the three thought conditions. Statistical significance for all analyses was set at $p < 0.05$. Of the three sessions, session 1 showed statistical significance. The decision performance of each thought condition was as follows: UT score = 3.57 (*SD* = 2.62); CT score = 2.14 (*SD* = 2.44); ID score = 1.43 (*SD* = 2.62). Because of significant UT effectiveness, session 1 was recognized as an appropriate stimulus: significant one-way ANOVA; $F$ (2, 26) = 4.24; $p < 0.05$. Therefore, we decided to use session 1 for the fMRI study.

## References


Strick, M., Dijksterhuis, A., and van Baaren, R.B. (2010). Unconscious-thought effects take place off-line, not on-line. *Psychological Science* 21(4)**,** 484-488.


## Figure legends

Figure 3. (Left) The letter “E” of the alphabet and (right) a real name in Japanese.
