# Supplementary figures and images for: Performance and Material-Dependent Holistic Representation of Unconscious Thought: A Functional Magnetic Resonance Imaging Study
Source: Front Hum Neurosci. 2019 Dec 6;13:418. doi: 10.3389/fnhum.2019.00418 (PMC6908964; doi:10.3389/fnhum.2019.00418)

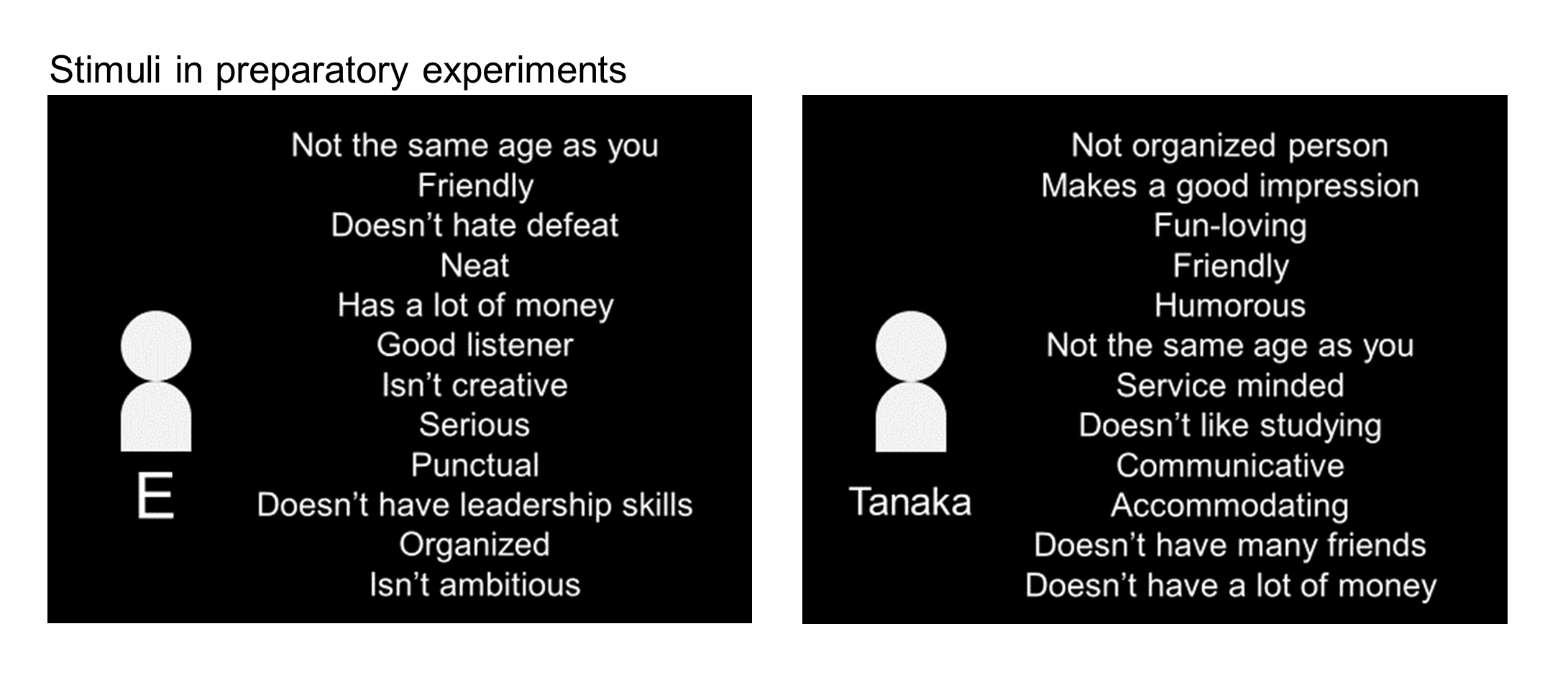

Supplement: Supplementary file 2 [file Image_1.tif]
